# Antipsychotic Prescribing for Behavioural Management in CAMHS: A Comparative Audit of Community and Inpatient Practice

**DOI:** 10.1192/bjo.2026.11729

**Published:** 2026-06-30

**Authors:** Nizar El Ahmadie, Stephen Hopkins, Chhaya Pandit

**Affiliations:** 1 Leeds and York Partnership NHS Foundation Trust, Leeds, United Kingdom; 2 Leeds Community Healthcare NHS Trust, Leeds, United Kingdom

## Abstract

**Aims:**

This audit evaluated antipsychotic prescribing practices for behavioural management in Child and Adolescent Mental Health Services (CAMHS) against National Institute for Health and Care Excellence (NICE) guidelines and local protocols. We hypothesised that prescribing indications would be guideline-compliant, while adherence to physical health monitoring would be lower in community settings compared to inpatient units. We further anticipated that the use of standardised outcome measures would be suboptimal across both services.

**Methods:**

A retrospective clinical audit was conducted on the electronic health records of 26 patients prescribed antipsychotics for behavioural management (13 from the Red Kite View inpatient unit; 13 from Leeds Community Healthcare community CAMHS). Data were collected between February 2023 and February 2025. Practice was measured against standards derived from NICE guidelines (NG11, CG155, CG170), the Maudsley Prescribing Guidelines, and local Trust protocols. Criteria included appropriateness of indication, specialist initiation, baseline and ongoing physical health monitoring, and the use of standardised outcome measures.

**Results:**

Compliance with indication and dosing guidelines was high, with 100% of cases documenting appropriate indications across both services. Specialist initiation was common (100% inpatient; 85% community). Documentation that psychological interventions were attempted prior to medication was lower in community settings (54%) compared with inpatient services (77%).

Significant deficits were identified in monitoring and outcome measurement. Standardised behavioural rating scales were not used in any cases across either setting (0%). Baseline movement disorder assessments were absent in community services (0%) and inconsistent in inpatient care (54%). Marked disparities were observed in baseline safety blood tests (full blood count, urea and electrolytes, liver function tests), completed in 92% of inpatient cases versus 38% in the community. Ongoing community monitoring demonstrated critical gaps, including 0% adherence to recommended weekly weight monitoring during titration and 0% completion of six-month liver function tests.

**Conclusion:**

The findings support the hypothesis: while clinical decision-making around antipsychotic initiation is largely guideline-compliant, substantial systemic gaps exist in physical health monitoring, particularly in community CAMHS. Furthermore, the universal absence of standardised outcome measures limits the objective assessment of benefit. Targeted interventions are required, including strengthened community physical health monitoring pathways, mandatory baseline movement disorder assessments, and the routine use of standardised behavioural outcome tools.

